# Supplementary material for: Acceptability and feasibility of video-based health education for maternal and infant health in Dirashe District, South Ethiopia: A qualitative study
Source: PLOS Glob Public Health. 2023 Jun 29;3(6):e0000821. doi: 10.1371/journal.pgph.0000821 (PMC10309618; doi:10.1371/journal.pgph.0000821)
Supplement: S1 Checklist — (DOCX) [file pgph.0000821.s001.docx]

**S1 COREQ Checklist for reporting qualitative studies**

| SNo | Items | Guiding questions/Description | Remark |
| --- | --- | --- | --- |
|  | **Domain 1: Research team and reflexivity** | |  |
|  | ***Personal characteristics*** | |  |
|  | Interviewer/facilitator | GY, KT conducted the interview and FGDs |  |
|  | Credentials | MSc, PhD, MD/PhD* | *SDH |
|  | Occupation | Researchers and academician |  |
|  | Gender | Both involved |  |
|  | Experience and training | Special training on qualitative data collection |  |
|  | ***Relationship with participant*** |  |  |
|  | Relationship established | Relations established |  |
|  | Participant knowledge of interviewer | The participants know about the interviewer as project team |  |
|  | Interviewer characteristics | Interest in the research topic considered and reported |  |
|  | **Domain 2: Study Design** |  |  |
|  | ***Theoretical framework*** |  |  |
|  | Methodological orientation and theory | Phenomenology design used |  |
|  | Sampling | Purposive sampling was used |  |
|  | Method of approaches | Face to face data collection was used |  |
|  | Sample size | 99(58person/FGDs, 41KII) |  |
|  | Non participation | 2 | 2mothers missed FGDs |
|  | ***Setting*** |  |  |
|  | Setting of data collection | Data collected at community health post with optimal seating |  |
|  | Presence of non-participants | non-participants not presented |  |
|  | Description of sample | All the interviews are with women aged 22 to 50 years |  |
|  | ***Data collection*** |  |  |
|  | Interview guide | Pretested interview guide was used |  |
|  | Repeat interview | Repeat interview not conducted |  |
|  | Audio/visual recording | Audio recording was used |  |
|  | Field note | Field notes were used to complement audio records |  |
|  | Duration | FGDs 1:25-1:45hour and KII last 35-55 minutes |  |
|  | Data saturation | Data saturated explained |  |
|  | Transcript returned | No | Due to security reasons after the translation of transcripts were not returned to participants |
|  | **Domain 3: Analysis and finding** |  |  |
|  | ***Data analysis*** |  |  |
|  | Number of data coders | Two data encoders code the data |  |
|  | Description of coding tree | Process of coding described |  |
|  | Derivation of theme | Sequential derivation of theme discussed |  |
|  | Software | Quirkos 2.1 |  |
|  | Participant checking | No | Due to security reasons after the translation of transcripts were not returned to participants |
|  | ***Reporting*** |  |  |
|  | Quotations presented | Quotations presented to support finding |  |
|  | Data and finding consistent | Yes |  |
|  | Clarity of major themes | Themes are defined and discussed |  |
|  | Clarity of minor themes | Minor themes are defined and discussed |  |
