# Supplementary material for: Acceptability and feasibility of video-based health education for maternal and infant health in Dirashe District, South Ethiopia: A qualitative study
Source: PLOS Glob Public Health. 2023 Jun 29;3(6):e0000821. doi: 10.1371/journal.pgph.0000821 (PMC10309618; doi:10.1371/journal.pgph.0000821)
Supplement: S1 File — (HTML) [file pgph.0000821.s002.html]

Effects of Video-based Health Education on Maternal and Child Health in Ethiopia - Tabular View - ClinicalTrials.gov


Try the modernized ClinicalTrials.gov beta website.
Learn more about the modernization effort.


Hide glossary

#### Glossary

Study record managers: refer to the Data Element Definitions if submitting registration or results information.

Search for terms


- Accepts healthy volunteers

  A type of eligibility criteria that indicates whether people who do not have the condition/disease being studied can participate in that clinical study.
- Active comparator arm

  An arm type in which a group of participants receives an intervention/treatment considered to be effective (or active) by health care providers.
- Adverse event

  An unfavorable change in the health of a participant, including abnormal laboratory findings, that happens during a clinical study or within a certain amount of time after the study has ended. This change may or may not be caused by the intervention/treatment being studied.
- Age or age group

  A type of eligibility criteria that indicates the age a person must be to participate in a clinical study. This may be indicated by a specific age or the following age groups:

  The age groups are:

  - Child (birth-17)
  - Adult (18-64)
  - Older Adult (65+)
- All-cause mortality

  A measure of all deaths, due to any cause, that occur during a clinical study.
- Allocation

  A method used to assign participants to an arm of a clinical study. The types of allocation are randomized allocation and nonrandomized.
- Arm

  A group or subgroup of participants in a clinical trial that receives a specific intervention/treatment, or no intervention, according to the trial's protocol.
- Arm type

  A general description of the clinical trial arm. It identifies the role of the intervention that participants receive. Types of arms include experimental arm, active comparator arm, placebo comparator arm, sham comparator arm, and no intervention arm.
- Baseline characteristics

  Data collected at the beginning of a clinical study for all participants and for each arm or comparison group. These data include demographics, such as age, sex/gender, race and ethnicity, and study-specific measures (for example, systolic blood pressure, prior antidepressant treatment).
- Canceled submission

  Indicates that the study sponsor or investigator recalled a submission of study results before quality control (QC) review took place. If the submission was canceled on or after May 8, 2018, the date is shown. After submission of study results, a study record cannot be modified until QC review is completed, unless the submission is canceled.
- Certain agreements

  Information required by the Food and Drug Administration Amendments Act of 2007. In general, this is a description of any agreement between the sponsor of a clinical study and the principal investigator (PI) that does not allow the PI to discuss the results of the study or publish the study results in a scientific or academic journal after the study is completed.
- Certification

  A sponsor or investigator may submit a certification to delay submission of results information if they are applying for FDA approval of a new drug or device, or new use of an already approved drug or device. A sponsor or investigator who submits a certification can delay results submission up to 2 years after the certification/extension first submitted date, unless certain events occur sooner. See Delay Results Type in the Results Data Element definitions for more information about this certification.
- Certification/extension first posted

  The date on which information about a certification to delay submission of results or an extension request was first available on ClinicalTrials.gov. ClinicalTrials.gov does not indicate whether the submission was a certification or extension request. There is typically a delay between the date the study sponsor or investigator submitted the certification or extension request and the first posted date.
- Certification/extension first submitted

  The date on which the study sponsor or investigator first submitted a certification or an extension request to delay submission of results. A sponsor or investigator who submits a certification can delay results submission up to 2 years after this date, unless certain events occur sooner. There is typically a delay between the date the certification or extension request was submitted and the date the information is first available on ClinicalTrials.gov (certification/extension first posted).
- Certification/extension first submitted that met QC criteria

  The date on which the study sponsor or investigator first submitted a certification or an extension request that is consistent with National Library of Medicine (NLM) quality control (QC) review criteria. The sponsor or investigator may need to revise and submit a certification or extension request one or more times before NLM's QC review criteria are met. It is the responsibility of the sponsor or investigator to ensure that the study record is consistent with the NLM QC review criteria. Meeting QC criteria for an extension request does not mean that the National Institutes of Health (NIH) has determined that the request demonstrates good cause. The process for review and granting of extension requests by the NIH is being developed.
- City and distance

  In the search feature, the City field is used to find clinical studies with locations in a specific city. The Distance field is used to find studies with locations within the specified distance from a city in number of miles. For example, if you choose Illinois as the state, identifying "Chicago" as the city and "100 miles" as the distance will find all studies listing a location within 100 miles of Chicago.
- Clinical study

  A research study involving human volunteers (also called participants) that is intended to add to medical knowledge. There are two types of clinical studies: interventional studies (also called clinical trials) and observational studies.
- Clinical trial

  Another name for an interventional study.
- ClinicalTrials.gov identifier (NCT number)

  The unique identification code given to each clinical study upon registration at ClinicalTrials.gov. The format is "NCT" followed by an 8-digit number (for example, NCT00000419).
- Collaborator

  An organization other than the sponsor that provides support for a clinical study. This support may include activities related to funding, design, implementation, data analysis, or reporting.
- Condition/disease

  The disease, disorder, syndrome, illness, or injury that is being studied. On ClinicalTrials.gov, conditions may also include other health-related issues, such as lifespan, quality of life, and health risks.
- Contact

  The name and contact information for the person who can answer enrollment questions for a clinical study. Each location where the study is being conducted may also have a specific contact, who may be better able to answer those questions.
- Country

  In the search feature, the Country field is used to find clinical studies with locations in a specific country. For example, if you choose the United States, you can then narrow your search by selecting a state and identifying a city and distance.
- Cross-over assignment

  A type of intervention model describing a clinical trial in which groups of participants receive two or more interventions in a specific order. For example, two-by-two cross-over assignment involves two groups of participants. One group receives drug A during the initial phase of the trial, followed by drug B during a later phase. The other group receives drug B during the initial phase, followed by drug A. So during the trial, participants "cross over" to the other drug. All participants receive drug A and drug B at some point during the trial but in a different order, depending on the group to which they are assigned.
- Data Monitoring Committee (DMC)

  A group of independent scientists who monitor the safety and scientific integrity of a clinical trial. The DMC can recommend to the sponsor that the trial be stopped if it is not effective, is harming participants, or is unlikely to serve its scientific purpose. Members are chosen based on the scientific skills and knowledge needed to monitor the particular trial. Also called a data safety and monitoring board, or DSMB.
- Early Phase 1 (formerly listed as Phase 0)

  A phase of research used to describe exploratory trials conducted before traditional phase 1 trials to investigate how or whether a drug affects the body. They involve very limited human exposure to the drug and have no therapeutic or diagnostic goals (for example, screening studies, microdose studies).
- Eligibility criteria

  The key requirements that people who want to participate in a clinical study must meet or the characteristics they must have. Eligibility criteria consist of both inclusion criteria (which are required for a person to participate in the study) and exclusion criteria (which prevent a person from participating). Types of eligibility criteria include whether a study  accepts healthy volunteers, has age or age group requirements, or is limited by sex.
- Enrollment

  The number of participants in a clinical study. The "estimated" enrollment is the target number of participants that the researchers need for the study.
- Exclusion criteria

  A type of eligibility criteria. These are reasons that a person is not allowed to participate in a clinical study.
- Expanded access

  A way for patients with serious diseases or conditions who cannot participate in a clinical trial to gain access to a medical product that has not been approved by the U.S. Food and Drug Administration (FDA). Also called compassionate use. There are different expanded access types.

  For more information, see FDA Expanded Access: Information for Patients.
- Expanded access status

  - **Available:** Expanded access is currently available for this investigational treatment, and patients who are not participants in the clinical study may be able to gain access to the drug, biologic, or medical device being studied.
  - **No longer available:** Expanded access was available for this intervention previously but is not currently available and will not be available in the future.
  - **Temporarily not available:** Expanded access is not currently available for this intervention but is expected to be available in the future.
  - **Approved for marketing:** The intervention has been approved by the  U.S. Food and Drug Administration for use by the public.
- Expanded access type

  Describes the category of expanded access under U.S. Food and Drug Administration (FDA) regulations. There are three types of expanded access:
  - **Individual Patients**: Allows a single patient, with a serious disease or condition who cannot participate in a clinical trial, access to a drug or biological product that has not been approved by the FDA. This category also includes access in an emergency situation.
  - **Intermediate-size Population**: Allows more than one patient (but generally fewer patients than through a Treatment IND/Protocol) access to a drug or biological product that has not been approved by the FDA. This type of expanded access is used when multiple patients with the same disease or condition seek access to a specific drug or biological product that has not been approved by the FDA.
  - **Treatment IND/Protocol**: Allows a large, widespread population access to a drug or biological product that has not been approved by the FDA. This type of expanded access can only be provided if the product is already being developed for marketing for the same use as the expanded access use.
- Experimental arm

  An arm type in which a group of participants receives the intervention/treatment that is the focus of the clinical trial.
- Extension request

  In certain circumstances, a sponsor or investigator may request an extension to delay the standard results submission deadline (generally one year after the primary completion date). The request for an extension must demonstrate good cause (for example, the need to preserve the scientific integrity of an ongoing masked trial). All requests must be reviewed and granted by the National Institutes of Health. This process for review and granting of extension requests is being developed. See Delay Results Type in the Results Data Element definitions for more information.
- Factorial assignment

  A type of intervention model describing a clinical trial in which groups of participants receive one of several combinations of interventions. For example, two-by-two factorial assignment involves four groups of participants. Each group receives one of the following pairs of interventions: (1) drug A and drug B, (2) drug A and a placebo, (3) a placebo and drug B, or (4) a placebo and a placebo. So during the trial, all possible combinations of the two drugs (A and B) and the placebos are given to different groups of participants.
- FDAAA 801 Violations

  A FDAAA 801 Violation is shown on a study record when the U.S. Food and Drug Administration (FDA) has issued a Notice of Noncompliance to the responsible party of an applicable clinical trial. A Notice of Noncompliance indicates that the FDA has determined the responsible party was not in compliance with the registration or results reporting requirements for the clinical trial under the Food and Drug Administration Amendments Act of 2007, Section 801 (FDAAA 801).

  The National Library of Medicine (NLM) is required by FDAAA 801 to add information to a study record about any FDAAA 801 Violation. This information is provided by the FDA. There are three categories of information that may be included:

  - Violation: Shown when the FDA issues a Notice of Noncompliance and posts the Notice of Noncompliance on its designated webpage. There are three types of violations:
    - Failure to submit required clinical trial information
    - Submission of false or misleading clinical trial information
    - Failure to submit primary and secondary outcomes
  - Correction: Shown when the FDA confirms that the responsible party has updated the study record to correct the violation and posts the correction notice on its designated webpage. Because of the time for FDA review and processing, there may be a delay between the date when the study record was updated and the addition of correction information to the FDAAA 801 Violation information.
  - Penalty: Shown when the FDA imposes a penalty for the violation and posts the penalty notice on its designated webpage.
- First posted

  The date on which the study record was first available on ClinicalTrials.gov after National Library of Medicine (NLM) quality control (QC) review has concluded. There is typically a delay of a few days between the date the study sponsor or investigator submitted the study record and the first posted date.
- First submitted

  The date on which the study sponsor or investigator first submitted a study record to ClinicalTrials.gov. There is typically a delay of a few days between the first submitted date and the record's availability on ClinicalTrials.gov (the first posted date).
- First submitted that met QC criteria

  The date on which the study sponsor or investigator first submits a study record that is consistent with National Library of Medicine (NLM) quality control (QC) review criteria. The sponsor or investigator may need to revise and submit a study record one or more times before NLM's QC review criteria are met. It is the responsibility of the sponsor or investigator to ensure that the study record is consistent with the NLM QC review criteria.
- Food and Drug Administration Amendments Act of 2007, Section 801 (FDAAA 801)

  U.S. Public Law 110-85, which was enacted on September 27, 2007. Section 801 of FDAAA amends Section 402 of the U.S. Public Health Service Act to expand ClinicalTrials.gov and create a clinical study results database. For more information on FDAAA 801, see the History, Policies, and Laws page on this site.
- Funder type

  Describes the organization that provides funding or support for a clinical study. This support may include activities related to funding, design, implementation, data analysis, or reporting. Organizations listed as sponsors and collaborators for a study are considered the funders of the study. ClinicalTrials.gov refers to four types of funders:
  - U.S. National Institutes of Health
  - Other U.S. Federal agencies (for example, Food and Drug Administration, Centers for Disease Control and Prevention, or U.S. Department of Veterans Affairs)
  - Industry (for example: pharmaceutical and device companies)
  - All others (including individuals, universities, and community-based organizations)
- Gender-based eligibility

  A type of eligibility criteria that indicates whether eligibility to participate in a clinical study is based a person's self-representation of gender identity or gender (yes, no). Gender is distinct from sex.
- Group/cohort

  A group or subgroup of participants in an observational study that is assessed for biomedical or health outcomes.
- Human subjects protection review board

  A group of people who review, approve, and monitor the clinical study's protocol. Their role is to protect the rights and welfare of people participating in a study (referred to as human research subjects), such as reviewing the informed consent form. The group typically includes people with varying backgrounds, including a community member, to make sure that research activities conducted by an organization are completely and adequately reviewed. Also called an institutional review board, or IRB, or an ethics committee.

  For more information, see Participating in Studies on this site.
- Inclusion criteria

  A type of eligibility criteria. These are the reasons that a person is allowed to participate in a clinical study.
- Informed consent

  A process used by researchers to communicate to potential and enrolled participants the risks and potential benefits of participating in a clinical study.

  For more information, see Participating in Studies on this site.
- Informed consent form (ICF)

  The document used in the informed consent or process.
- Intervention model

  The general design of the strategy for assigning interventions to participants in a clinical study. Types of intervention models include: single group assignment, parallel assignment, cross-over assignment, and factorial assignment.
- Intervention/treatment

  A process or action that is the focus of a clinical study. Interventions include drugs, medical devices, procedures, vaccines, and other products that are either investigational or already available. Interventions can also include noninvasive approaches, such as education or modifying diet and exercise.
- Interventional study (clinical trial)

  A type of clinical study in which participants are assigned to groups that receive one or more intervention/treatment (or no intervention) so that researchers can evaluate the effects of the interventions on biomedical or health-related outcomes. The assignments are determined by the study's protocol. Participants may receive diagnostic, therapeutic, or other types of interventions.
- Investigator

  A researcher involved in a clinical study. Related terms include site principal investigator, site sub-investigator, study chair, study director, and study principal investigator.
- Last update posted

  The most recent date on which changes to a study record were made available on ClinicalTrials.gov. There may be a delay between when the changes were submitted to ClinicalTrials.gov by the study's sponsor or investigator (the last update submitted date) and the last update posted date.
- Last update submitted

  The most recent date on which the study sponsor or investigator submitted changes to a study record to ClinicalTrials.gov. There is typically a delay of a few days between the last update submitted date and when the date changes are posted on ClinicalTrials.gov (the last update posted date).
- Last update submitted that met QC criteria

  The most recent date on which the study sponsor or investigator submitted changes to a study record that are consistent with National Library of Medicine (NLM) quality control (QC) review criteria. It is the responsibility of the sponsor or investigator to ensure that the study record is consistent with the NLM QC review criteria.
- Last verified

  The most recent date on which the study sponsor or investigator confirmed the information about a clinical study on ClinicalTrials.gov as accurate and current. If a study with a recruitment status of recruiting; not yet recruiting; or active, not recruiting has not been confirmed within the past 2 years, the study's recruitment status is shown as unknown.
- Listed location countries

  Countries in which research facilities for a study are located. A country is listed only once, even if there is more than one facility in the country. The list includes all countries as of the last update submitted date; any country for which all facilities were removed from the study record are listed under removed location countries.
- Location terms

  In the search feature, the Location terms field is used to narrow a search by location-related terms other than Country, State, and City or distance. For example, you may enter a specific facility name (such as National Institutes of Health Clinical Center) or a part of a facility name (such as Veteran for studies listing Veterans Hospital or Veteran Affairs in the facility name). Note: Not all study records include this level of detail about locations.
- Masking

  A clinical trial design strategy in which one or more parties involved in the trial, such as the investigator or participants, do not know which participants have been assigned which interventions. Types of masking include: open label, single blind masking, and double-blind masking.
- NCT number

  A unique identification code given to each clinical study record registered on ClinicalTrials.gov. The format is "NCT" followed by an 8-digit number (for example, NCT00000419). Also called the ClinicalTrials.gov identifier.
- No intervention arm

  An arm type in which a group of participants does not receive any intervention/treatment during the clinical trial.
- Observational study

  A type of clinical study in which participants are identified as belonging to study groups and are assessed for biomedical or health outcomes. Participants may receive diagnostic, therapeutic, or other types of interventions, but the investigator does not assign participants to a specific interventions/treatment.

  A patient registry is a type of observational study.
- Observational study model

  The general design of the strategy for identifying and following up with participants during an observational study. Types of observational study models include cohort, case-control, case-only, case-cross-over, ecologic or community studies, family-based, and other.
- Other adverse event

  An adverse event that is not a serious adverse event, meaning that it does not result in death, is not life-threatening, does not require inpatient hospitalization or extend a current hospital stay, does not result in an ongoing or significant incapacity or interfere substantially with normal life functions, and does not cause a congenital anomaly or birth defect; it also does not put the participant in danger and does not require medical or surgical intervention to prevent one of the results listed above.
- Other study IDs

  Identifiers or ID numbers other than the NCT number that are assigned to a clinical study by the study's sponsor, funders, or others. These numbers may include unique identifiers from other trial registries and National Institutes of Health grant numbers.
- Other terms

  In the search feature, the Other terms field is used to narrow a search. For example, you may enter the name of a drug or the NCT number of a clinical study to limit the search to study records that contain these words.
- Outcome measure

  For clinical trials, a planned measurement described in the protocol that is used to determine the effect of an intervention/treatment on participants. For observational studies, a measurement or observation that is used to describe patterns of diseases or traits, or associations with exposures, risk factors, or treatment. Types of outcome measures include primary outcome measure and secondary outcome measure.
- Parallel assignment

  A type of intervention model describing a clinical trial in which two or more groups of participants receive different interventions. For example, a two-arm parallel assignment involves two groups of participants. One group receives drug A, and the other group receives drug B. So during the trial, participants in one group receive drug A "in parallel" to participants in the other group, who receive drug B.
- Participant flow

  A summary of the progress of participants through each stage of a clinical study, by study arm or group/cohort. This includes the number of participants who started, completed, and dropped out of the study.
- Patient registry

  A type of observational study that collects information about patients' medical conditions and/or treatments to better understand how a condition or treatment affects patients in the real world.
- Phase

  The stage of a clinical trial studying a drug or biological product, based on definitions developed by the U.S. Food and Drug Administration (FDA). The phase is based on the study's objective, the number of participants, and other characteristics. There are five phases: Early Phase 1 (formerly listed as Phase 0), Phase 1, Phase 2, Phase 3, and Phase 4. Not Applicable is used to describe trials without FDA-defined phases, including trials of devices or behavioral interventions.
- Phase 1

  A phase of research to describe clinical trials that focus on the safety of a drug. They are usually conducted with healthy volunteers, and the goal is to determine the drug's most frequent and serious adverse events and, often, how the drug is broken down and excreted by the body. These trials usually involve a small number of participants.
- Phase 2

  A phase of research to describe clinical trials that gather preliminary data on whether a drug works in people who have a certain condition/disease (that is, the drug's effectiveness). For example, participants receiving the drug may be compared to similar participants receiving a different treatment, usually an inactive substance (called a placebo) or a different drug. Safety continues to be evaluated, and short-term adverse events are studied.
- Phase 3

  A phase of research to describe clinical trials that gather more information about a drug's safety and effectiveness by studying different populations and different dosages and by using the drug in combination with other drugs. These studies typically involve more participants.
- Phase 4

  A phase of research to describe clinical trials occurring after FDA has approved a drug for marketing. They include postmarket requirement and commitment studies that are required of or agreed to by the study sponsor. These trials gather additional information about a drug's safety, efficacy, or optimal use.
- Phase Not Applicable

  Describes trials without FDA-defined phases, including trials of devices or behavioral interventions.
- Placebo

  An inactive substance or treatment that looks the same as, and is given in the same way as, an active drug or intervention/treatment being studied.
- Placebo comparator arm

  An arm type in which a group of participants receives a placebo during a clinical trial.
- Primary completion date

  The date on which the last participant in a clinical study was examined or received an intervention to collect final data for the primary outcome measure. Whether the clinical study ended according to the protocol or was terminated does not affect this date. For clinical studies with more than one primary outcome measure with different completion dates, this term refers to the date on which data collection is completed for all the primary outcome measures. The "estimated" primary completion date is the date that the researchers think will be the primary completion date for the study.
- Primary outcome measure

  In a clinical study's protocol, the planned outcome measure that is the most important for evaluating the effect of an intervention/treatment. Most clinical studies have one primary outcome measure, but some have more than one.
- Primary purpose

  The main reason for the clinical trial. The types of primary purpose are: treatment, prevention, diagnostic, supportive care, screening, health services research, basic science, and other.
- Principal investigator (PI)

  The person who is responsible for the scientific and technical direction of the entire clinical study.
- Protocol

  The written description of a clinical study. It includes the study's objectives, design, and methods. It may also include relevant scientific background and statistical information.
- Quality control (QC) review

  National Library of Medicine (NLM) staff perform a limited review of submitted study records for apparent errors, deficiencies, or inconsistencies. NLM staff identify potential major and advisory issues and provide comments directly to the study sponsor or investigator. Major issues identified in QC review must be addressed or corrected (see First submitted that met QC criteria and Results first submitted that met QC criteria). Advisory issues are suggestions to help improve the clarity of the record. NLM staff do not verify the scientific validity or relevance of the submitted information. The study sponsor or investigator is responsible for ensuring that the studies follow all applicable laws and regulations.
- Randomized allocation

  A type of allocation strategy in which participants are assigned to the arms of a clinical trial by chance.
- Recruitment status

  - **Not yet recruiting:** The study has not started recruiting participants.
  - **Recruiting:** The study is currently recruiting participants.
  - **Enrolling by invitation:** The study is selecting its participants from a population, or group of people, decided on by the researchers in advance. These studies are not open to everyone who meets the eligibility criteria but only to people in that particular population, who are specifically invited to participate.
  - **Active, not recruiting:** The study is ongoing, and participants are receiving an intervention or being examined, but potential participants are not currently being recruited or enrolled.
  - **Suspended:** The study has stopped early but may start again.
  - **Terminated:** The study has stopped early and will not start again. Participants are no longer being examined or treated.
  - **Completed:** The study has ended normally, and participants are no longer being examined or treated (that is, the last participant's last visit has occurred).
  - **Withdrawn:** The study stopped early, before enrolling its first participant.
  - **Unknown:** A study on ClinicalTrials.gov whose last known status was recruiting; not yet recruiting; or active, not recruiting but that has passed its completion date, and the status has not been last verified within the past 2 years.
- Registration

  The process of submitting and updating summary information about a clinical study and its protocol, from its beginning to end, to a structured, public Web-based study registry that is accessible to the public, such as ClinicalTrials.gov.
- Removed location countries

  Countries that appeared under listed location countries but were removed from the study record by the sponsor or investigator.
- Reporting group

  A grouping of participants in a clinical study that is used for summarizing the data collected during the study. This grouping may be the same as or different from a study arm or group.
- Responsible party

  The person responsible for submitting information about a clinical study to ClinicalTrials.gov and updating that information. Usually the study sponsor or investigator.
- Results database

  A structured online system, such as the ClinicalTrials.gov results database, that provides the public with access to registration and summary results information for completed or terminated clinical studies. A study with results available on ClinicalTrials.gov is described as having the results "posted."

  **Note:** The ClinicalTrials.gov results database became available in September 2008. Older studies are unlikely to have results available in the database.
- Results delayed

  Indicates that the sponsor or investigator submitted a certification or extension request.
- Results first posted

  The date on which summary results information was first available on ClinicalTrials.gov after National Library of Medicine (NLM) quality control (QC) review has concluded. There is typically a delay between the date the study sponsor or investigator first submits summary results information (the results first submitted date) and the results first posted date. Some results information may be available at an earlier date if Results First Posted with QC Comments.
- Results first posted with QC comments

  The date on which summary results information was first available on ClinicalTrials.gov with quality control review comments from the National Library of Medicine (NLM) identifying major issues that must be addressed by the sponsor or investigator. As of January 1, 2020, initial results submissions for applicable clinical trials (ACTs) that do not meet quality control review criteria will be publicly posted on ClinicalTrials.gov with brief standardized major comments. Accordingly, the Results First Posted with QC Comments date may be earlier than the Results First Posted date for an ACT with summary results information that is not consistent with NLM quality control review criteria.
- Results first submitted

  The date on which the study sponsor or investigator first submits a study record with summary results information. There is typically a delay between the results first submitted date and when summary results information becomes available on ClinicalTrials.gov (the results first posted date).
- Results first submitted that met QC criteria

  The date on which the study sponsor or investigator first submits a study record with summary results information that is consistent with National Library of Medicine (NLM) quality control (QC) review criteria. The sponsor or investigator may need to revise and submit results information one or more times before NLM's QC review criteria are met. It is the responsibility of the sponsor or investigator to ensure that the study record is consistent with the NLM QC review criteria.
- Results returned after quality control review

  The date on which the National Library of Medicine provided quality control (QC) review comments to the study sponsor or investigator. The sponsor or investigator must address major issues identified in the review comments. If there is a date listed for results returned after quality control review, but there is not a subsequent date listed for results submitted to ClinicalTrials.gov, this means that the submission is pending changes by the sponsor or investigator.
- Results submitted to ClinicalTrials.gov

  Indicates that the study sponsor or investigator has submitted summary results information for a clinical study to ClinicalTrials.gov but the quality control (QC) review process has not concluded.

  The results submitted date indicates when the study sponsor or investigator first submitted summary results information or submitted changes to summary results information. Submissions with changes are typically in response to QC review comments from the National Library of Medicine (NLM). If there is a date listed for results submitted to ClinicalTrials.gov, but there is not a subsequent date listed for results returned after quality control review, this means that the submission is pending review by NLM.
- Secondary outcome measure

  In a clinical study's protocol, a planned outcome measure that is not as important as the primary outcome measure for evaluating the effect of an intervention but is still of interest. Most clinical studies have more than one secondary outcome measure.
- Serious adverse event

  An adverse event that results in death, is life-threatening, requires inpatient hospitalization or extends a current hospital stay, results in an ongoing or significant incapacity or interferes substantially with normal life functions, or causes a congenital anomaly or birth defect. Medical events that do not result in death, are not life-threatening, or do not require hospitalization may be considered serious adverse events if they put the participant in danger or require medical or surgical intervention to prevent one of the results listed above.
- Sex

  A type of eligibility criteria that indicates the sex of people who may participate in a clinical study (all, female, male). Sex is a person's classification as female or male based on biological distinctions. Sex is distinct from gender-based eligibility.
- Sham comparator arm

  An arm type in which a group of participants receives a procedure or device that appears to be the same as the actual procedure or device being studied but does not contain active processes or components.
- Single group assignment

  A type of intervention model describing a clinical trial in which all participants receive the same intervention/treatment.
- Sort studies by

  In Advanced Search, the Sort studies by option is used to change the order of studies listed on the Search Results page. You can sort by Relevance or Newest First:
  - Relevance: Studies that best match your search terms appear higher in the search results list. This is the default display for all searches.
  - Newest First: Studies with the most recent First posted dates appear higher in the search results list.
- Sponsor

  The organization or person who initiates the study and who has authority and control over the study.
- State

  In the search feature, the State field is used to find clinical studies with locations in a specific state within the United States. If you choose United States in the Country field, you can search for studies with locations in a specific state.
- Statistical analysis plan (SAP)

  The written description of the statistical considerations and methods for analyzing the data collected in the clinical study.
- Status

  Indicates the current recruitment status or the expanded access status.
- Study completion date

  The date on which the last participant in a clinical study was examined or received an intervention/treatment to collect final data for the primary outcome measures, secondary outcome measures, and adverse events (that is, the last participant's last visit). The "estimated" study completion date is the date that the researchers think will be the study completion date.
- Study design

  The investigative methods and strategies used in the clinical study.
- Study documents

  Refers to the type of documents that the study sponsor or principal investigator may add to their study record. These include a study protocol, statistical analysis plan, and informed consent form.
- Study IDs

  Identifiers that are assigned to a clinical study by the study's sponsor, funders, or others. They include unique identifiers from other trial study registries and National Institutes of Health grant numbers. Note: ClinicalTrials.gov assigns a unique identification code to each clinical study registered on ClinicalTrials.gov. Also called the NCT number, the format is "NCT" followed by an 8-digit number (for example, NCT00000419).
- Study record

  An entry on ClinicalTrials.gov that contains a summary of a clinical study's protocol information, including the recruitment status; eligibility criteria; contact information; and, in some cases, summary results. Each study record is assigned a ClinicalTrials.gov identifier, or NCT number.
- Study registry

  A structured online system, such as ClinicalTrials.gov, that provides the public with access to summary information about ongoing and completed clinical studies.
- Study results

  A study record that includes the summary results posted in the ClinicalTrials.gov results database. Summary results information includes participant flow, baseline characteristics, outcome measures, and adverse events (including serious adverse events).
- Study start date

  The actual date on which the first participant was enrolled in a clinical study. The "estimated" study start date is the date that the researchers think will be the study start date.
- Study type

  Describes the nature of a clinical study. Study types include interventional studies (also called clinical trials), observational studies (including patient registries), and expanded access.
- Submitted date

  The date on which the study sponsor or investigator submitted a study record that is consistent with National Library of Medicine (NLM) quality control (QC) review criteria.
- Title

  The official title of a protocol used to identify a clinical study or a short title written in language intended for the lay public.
- Title acronym

  The acronym or initials used to identify a clinical study (not all studies have one). For example, the title acronym for the Women's Health Initiative is "WHI."
- U.S. Agency for Healthcare Research and Quality (AHRQ)

  An agency within the U.S. Department of Health and Human Services. AHRQ's mission is to produce evidence to make health care safer, higher quality, more accessible, equitable, and affordable, and to work within the U.S. Department of Health and Human Services and with other partners to make sure that the evidence is understood and used.
- U.S. Food and Drug Administration (FDA)

  An agency within the U.S. Department of Health and Human Services. The FDA is responsible for protecting the public health by making sure that human and veterinary drugs, vaccines and other biological products, medical devices, the Nation's food supply, cosmetics, dietary supplements, and products that give off radiation are safe, effective, and secure.
- Unknown

  A type of recruitment status. It identifies a study on ClinicalTrials.gov whose last known status was recruiting; not yet recruiting; or active, not recruiting but that has passed its completion date, and the status has not been verified within the past 2 years. Studies with an unknown status are considered closed studies.

×

- Find Studies
  - New Search
  - Advanced Search
  - See Studies by Topic
  - See Studies on Map
  - How to Search
  - How to Use Search Results
  - How to Find Results of Studies
  - How to Read a Study Record
- About Studies
  - Learn About Studies
  - Other Sites About Studies
  - Glossary of Common Site Terms
- Submit Studies
  - Submit Studies to ClinicalTrials.gov PRS
  - Why Should I Register and Submit Results?
  - FDAAA 801 and the Final Rule
  - How to Apply for a PRS Account
  - How to Register Your Study
  - How to Edit Your Study Record
  - How to Submit Your Results
  - Frequently Asked Questions
  - Support Materials
  - Training Materials
- Resources
  - Selected Publications
  - Clinical Alerts and Advisories
  - RSS Feeds
  - Trends, Charts, and Maps
  - Downloading Content for Analysis
- About Site
  - What's New
  - ClinicalTrials.gov Background
  - About the Results Database
  - History, Policies, and Laws
  - ClinicalTrials.gov Modernization
  - Media/Press Resources
  - Linking to This Site
  - Terms and Conditions
  - Disclaimer
- PRS Login

- Find Studies
  - New Search
  - Advanced Search
  - See Studies by Topic
  - See Studies on Map
  - How to Search
  - How to Use Search Results
  - How to Find Results of Studies
  - How to Read a Study Record
- About Studies
  - Learn About Studies
  - Other Sites About Studies
  - Glossary of Common Site Terms
- Submit Studies
  - Submit Studies to ClinicalTrials.gov PRS
  - Why Should I Register and Submit Results?
  - FDAAA 801 and the Final Rule
  - How to Apply for a PRS Account
  - How to Register Your Study
  - How to Edit Your Study Record
  - How to Submit Your Results
  - Frequently Asked Questions
  - Support Materials
  - Training Materials
- Resources
  - Selected Publications
  - Clinical Alerts and Advisories
  - RSS Feeds
  - Trends, Charts, and Maps
  - Downloading Content for Analysis
- About Site
  - What's New
  - ClinicalTrials.gov Background
  - About the Results Database
  - History, Policies, and Laws
  - ClinicalTrials.gov Modernization
  - Media/Press Resources
  - Linking to This Site
  - Terms and Conditions
  - Disclaimer
- PRS Login

- Home
- Search Results
- Study Record Detail

Saved Studies (1)

Save this study

**Warning**

You have reached the maximum number of saved studies (100).

Please remove one or more studies before adding more.

# Effects of Video-based Health Education on Maternal and Child Health in Ethiopia (MCH)

|  |  |
| --- | --- |
|  | The safety and scientific validity of this study is the responsibility of the study sponsor and investigators. Listing a study does not mean it has been evaluated by the U.S. Federal Government. Read our disclaimer for details. |

|  |
| --- |
| ClinicalTrials.gov Identifier: NCT04414527 |
| Recruitment Status  : Completed First Posted  : June 4, 2020  Last Update Posted  : December 6, 2021 |

Sponsor:

University Ghent

Collaborators:

Flemish Interuniversity Council (VLIR)

College of Medicine and Health Sciences, Arba Minch University, Ethiopia

Information provided by (Responsible Party):

University Ghent

  

- Study Details
- Tabular View
- No Results Posted
- Disclaimer
- How to Read a Study Record

| Tracking Information | |
| --- | --- |
| First Submitted Date  ICMJE | April 21, 2020 |
| First Posted Date  ICMJE | June 4, 2020 |
| Last Update Posted Date | December 6, 2021 |
| Actual Study Start Date  ICMJE | March 13, 2020 |
| Actual Primary Completion Date | July 31, 2021   (Final data collection date for primary outcome measure) |
| Current Primary Outcome Measures  ICMJE    (submitted: May 29, 2020) | - Adherence to iron and folic acid supplementation during pregnancy [ Time Frame: Monthly during six months pregnancy ] Monthly disappearance rate of IFA tablets - Adherence to iron and folic acid supplementation post-partum [ Time Frame: Monthly during three months postpartum ] Monthly disappearance rate of IFA tablets - Maternal anemia during pregnancy [ Time Frame: Hemoglobin concentrations will be measured at 9 months pregnancy ] Hemoglobin concentrations (g/dL) - Maternal anemia post-partum [ Time Frame: Hemoglobin concentrations will be measured at six months postpartum ] Hemoglobin concentrations (g/dL) - Early initiation [ Time Frame: At birth (six months after the enrollment) ] Prevalence of newborns put to the breast in the first hour after birth - Exclusive breastfeeding [ Time Frame: Birth to six months postpartum ] Prevalence of infants exclusively breastfed using maternal reports and the deuterium dose-to-mother technique (in a subgroup) - Dietary intake during six months pregnancy [ Time Frame: Assessed at 6 months and 9 months pregnancy ] Prevalence of women with adequate dietary intake during six months pregnancy - Dietary intake at six months post-partum [ Time Frame: Assessed at six months postpartum ] Prevalence of women with adequate dietary intake at six months post-partum |
| Original Primary Outcome Measures  ICMJE | *Same as current* |
| Change History | Complete list of historical versions of study NCT04414527 on ClinicalTrials.gov Archive Site |
| Current Secondary Outcome Measures  ICMJE    (submitted: May 29, 2020) | - Gestational weight gain [ Time Frame: Gestational weight gain will be measured in all pregnant women at six and nine months pregnancy ] Weight gain at term (Kg) - Maternal genital infections [ Time Frame: Maternal genital infections will be assessed at nine months pregnancy ] The presence of genital infections that are known to affect a healthy pregnancy, including but not limited to bacterial vaginosis, Chlamydia trachomatis, Neisseria gonorrhoeae, Trichomonas vaginalis, Listeria monocytogenes. - Birth weight [ Time Frame: Birth weight will be assessed in all newborns ] Birth weight (g) - Infant weight [ Time Frame: Weight of infants will be assessed monthly from birth until six months of age ] Infant weight (g) on a monthly basis - Infant length [ Time Frame: Length of infants will be assessed monthly from birth until six months of age ] Infant length (cm) on a monthly basis - Infant anemia [ Time Frame: Hemoglobin concentrations will be measured at six months of age ] Hemoglobin concentrations (g/dL) - Maternal parasitic infections [ Time Frame: Worm infections will be assessed in all women at 6 months pregnancy, 9 months pregnancy, and at two weeks- and 6 months post partum ] The presence of worm parasites and egg density in the stools. Three common parasites and their eggs will be investigated, i.e. Ascaris lumbricoides (round worm), Trichuris trichiura (whipworm) and Ancyclostoma duodenale or Necater americanus (hookworms). - Infant parasitic infections [ Time Frame: Infant parasitic infections will be assessed at 6 months of age. ] The prevalence of Giarida and Cryposporidium will be assessed in all infants - Maternal plasma ferritin [ Time Frame: Plasma ferritin is assessed in a subgroup of women at 9 months pregnancy and at six months postpartum ] Iron status as indicated plasma ferritin (micro\_g/L) is a test to evaluate iron stores - Infant plasma ferritin [ Time Frame: Plasma ferritin is assessed in a subgroup of infants at six months of age ] Iron status as indicated plasma ferritin (micro\_g/L) is a test to evaluate iron stores - Maternal soluble transferrin receptor [ Time Frame: Plasma ferritin is assessed in a subgroup of women at 9 months pregnancy and at six months postpartum ] Soluble transferrin receptor (mg/L) is an indicator for iron deficiency especially in high inflammation settings - Infant soluble transferrin receptor [ Time Frame: Soluble transferrin receptor is assessed in a subgroup of infants at six months of age ] Soluble transferrin receptor (mg/L) is an indicator for iron deficiency especially in high inflammation settings - Maternal serum concentrations in Vitamin A (retinol) [ Time Frame: Serum concentrations in Vitamin A are assessed in a subgroup of women at 9 months pregnancy and at six months postpartum ] Retinol concentrations in serum is an indicator of vitamin A status - Infant serum concentrations in Vitamin A (retinol) [ Time Frame: Serum concentrations in Vitamin A are assessed in a subgroup of infants at six months of age ] Retinol concentrations in serum is an indicator of vitamin A status - Maternal serum concentrations in vitamin B12 [ Time Frame: Vitamin B12 concentrations will be assessed in a subgroup of women at 9 months pregnancy and at six months postpartum ] Serum concentrations in vitamin B12 - Infant serum concentrations in vitamin B12 [ Time Frame: Vitamin B12 concentrations will be assessed in a subgroup of infants at six months of age ] Serum concentrations in vitamin B12 |
| Original Secondary Outcome Measures  ICMJE | *Same as current* |
| Current Other Pre-specified Outcome Measures | Not Provided |
| Original Other Pre-specified Outcome Measures | Not Provided |
|  | |
| Descriptive Information | |
| Brief Title  ICMJE | Effects of Video-based Health Education on Maternal and Child Health in Ethiopia |
| Official Title  ICMJE | Effects of Video-based Health Education on Health Status of Pregnant Mothers and Their Infants (From 0 to 6 Months) in Dirashe District Southern Ethiopia - a Cluster Randomized Controlled Trial. |
| Brief Summary | Low adherence to recommended health and nutrition strategies during the critical 1000 day-window of opportunity is multifactorial but low quality communication is key limitation. Innovative strategies to improve interpersonal communication can reduce the burden and the fatigue of community health workers and may result in a greater change. The findings of this project will support governments and other stakeholders in their delivery of high impact nutrition and health practices.  This intervention aims to improve adherence to ante- and post-natal care practices and recommendations by the use of our video-based health education. These videos will be implemented through home-based counseling by trained assistants, and video-based forum participation led by community nurses and health extension workers (HEWs). During the monthly forums, the educational package will be delivered in a video form - locally prepared using multiple approaches like testimony, comedy, dramas in the form of questions and answers, group discussions and deductive approaches. Cordless projectors and locally created videos give the health community more quality control over the end message, expand the number of people reached, allow for the use of minimally trained non-expert facilitators such as the hews, and allow for contextually appropriate information. They can also be used in areas without access to electricity, helping to bridge the digital divide, and serving as a leapfrog technology for areas that would otherwise not have access to media. |
| Detailed Description | In Ante- and post-natal care, low adherence to recommended health and nutrition strategies during the critical 1,000 day-window of opportunity is multifactorial, but low-quality communication is key limitation. Innovative strategies to improve interpersonal communication can reduce the burden and the fatigue of community health workers and may result in a greater change. The findings of this project will support governments and other stakeholders in their delivery of high impact nutrition and health practices.  Focused antenatal care (FANC), including iron and folic acid supplementation (IFA) is one of the main strategies to reduce maternal and child deaths. The WHO recommends at least four hospital visits during the pregnancy. Supplementation with IFA during pregnancy improves birth weight and reduces megaloblastic anemia by 79%. The uptake of nutritional and health practices is influenced by complex, contextual determinants at the individual and community levels. Evidence showed that Social and Behavior Change Communication (SBCC) is an effective approach to increase the uptake of key strategies and to sustain behavior change.  Ethiopia, a low-income country in sub-Saharan Africa, has one of the highest maternal and infant mortality rates. It is estimated that 676 mothers die per 100,000 live births and that 59 infants die per 1,000 live births. Maternal anemia is associated with an increased risk of maternal death. Iron deficiency anemia is a strong risk factor for low birthweight (LBW) and perinatal mortality. genital infections such as bacterial vaginosis, candidiasis and worm infections (such as intestinal hookworm infections) are considered important infections that possibly could confound the study results. Bacterial vaginosis and candidiasis are a known risk factor for preterm birth. these infections are also linked with anemia and maternal nutritional status. Hookworm infections are highly prevalent in Ethiopia and are associated with undernutrition and anemia.  The reports of the Ethiopian Demographic and Health Survey showed an increase in women aged 15-49 years in Ethiopia receiving antenatal care (ANC) from a skilled provider up to 62% in 2016 (EDHS, 2016). The percentage of women taking IFA supplements for 90 days or more remains at a substandard level of only 5% (EDHS, 2016). Antenatal care coverage for at least one visit is 28% but coverage for at least four visits declines to 12%, suggesting systemic barriers that potentially prevent the mothers from returning to the health centers. One of the barriers may be perceived failure of the existing interventions to make a meaningful impact that could stimulate the desired behavioral change.  This intervention aims to improve adherence to ante- and post-natal care practices and recommendations by the use of our video-based health education. These videos will be implemented through home-based counseling by trained assistants, and video-based forum participation led by community nurses and Health Extension Workers (HEWs). The nutrition-specific education packages will be based on the WHO-UNICEF key messages booklet on the community, infant and young child feeding counseling package and will be culturally adjusted to fit the local context and translated into the main four local languages. The videos will also include some hygienic aspects that reduce the risk of both genital and parasitic infections, that are also causing undernutrition, anemia and/or adverse pregnancy outcomes. During the monthly forums, the educational package will be delivered in a video form - locally prepared using multiple approaches like testimony, comedy, dramas in the form of questions and answers, group discussions and deductive approaches (more details can be found on OMPT website https://www.ompt.org/).  The main objective of this project is to assess the effects of this innovative video-based health education on reproductive health, and on birth outcomes and the nutritional status of women and their infants from birth to six months of age.  PRIMARY OBJECTIVES   1. To assess the effects of video-based health education package provided to pregnant and lactating women on the knowledge, attitude and practice on recommended health including adherence to ANC visits and to IFA supplementation. 2. To assess the effects of video-based health education on birth outcomes and anemia status of women during pregnancy, at delivery and six-month postpartum. 3. To evaluate the effect of video-based health education on early initiation and exclusive breastfeeding (EBF) of infants from 0-6 months of age In this two-arm cluster randomized trial, 675 pregnant women in their first trimester (12 weeks of gestation) will be recruited and followed up until delivery and then with their infants for six months postpartum.   The intervention will include home to home visit for delivery of healthy nutrition and hygienic messages using prepared video-based messages. participation in monthly forums will be facilitated by nurses using also videos for demonstration of nutritional and hygienic care and will be delivered at the homes of the participants every month by trained HEW until delivery, in addition to the ANC regular visits. During the monthly forums (six in total during the pregnancy and the post-partum periods), the messages will all be given as a video show, coordinated by a nurse/ health professional who will further answer any questions. During the postnatal period, two counseling sessions will be organized within the first two weeks after birth, and a further six sessions ( every month) till 6 months postnatally.  The HEW will distribute the IFA 30 tablets (30 mg elemental iron and 400 µg of folic acid) every month, and will provide counseling on the importance of- and instructions on adherence and other recommendations as detailed earlier. Pregnant women in the control group will receive the standard education package as per the Ethiopian guidelines. In the standard health care, pregnant women receive a minimum of four ANC visits at the health centers during which they also receive IFA supplementation. The control and the intervention groups receive the same amount of tablets (i.e. 30 tablets containing 30 mg elemental iron and 400 µg of folic acid, every month). Monthly IFA utilization will be checked through HEW or our trained service delivery workers during home to home visit. Women who test positive for soil-transmitted helminth will be treated according to the national protocol starting from the second trimester (treatment is not advised during the first trimester). Women who experience odor, itching or discharge will be treated for candidiasis and bacterial vaginosis.  Data will be collected in pregnant women at baseline, at six and at 9 months of pregnancy . After delivery data will be collected in the pairs mother-infant within two weeks and at 3 and 6 months postpartum. At the different time points, biological samples will be collected to assess the micronutrient status, the presence of inflammations and the presence of genital and parasitic infections. |
| Study Type  ICMJE | Interventional |
| Study Phase  ICMJE | Not Applicable |
| Study Design  ICMJE | Allocation: Randomized Intervention Model: Parallel Assignment Intervention Model Description: In a two-arm cluster randomized trial, 675 eligible pregnant women in their first trimester (12 weeks of gestation) will be recruited and followed up until delivery, and in pair with their infant up-to six months postpartum.   1. Eligible pregnant women in the intervention group will receive video-based nutritional and hygienic education package (Health-Video) for six months until delivery and then for another six-month postpartum. 2. Pregnant women in the control group will receive the standard education package as per the Ethiopian guidelines.   The intervention will include home-to-home visit for delivery of healthy nutrition and hygiene messages using prepared video-based messages, and participation in monthly forums facilitated by nurses using also videos for demonstration of nutritional and hygienic care. Masking: None (Open Label) Primary Purpose: Prevention |
| Condition  ICMJE | - Anemia - Antenatal Care - Birth Outcomes - Worm Infection - Bacterial Vaginoses - Exclusive Breastfeeding |
| Intervention  ICMJE | - Behavioral: Standard counselling The control cohort will receive national standard counseling during four ante-natal care visits.  Women in the control group will receive additionally    1. national nutrition and health care including IFA supplementation,   2. treatment of any symptomatic health condition and deworming in case of symptomatic complaints during second and third trimesters,   3. Women who experience odor, itching or discharge will be treated for candidiasis and bacterial vaginosis.  Other Name: Control - Behavioral: Health-Video The intervention cohort Health-Video will receive innovative video-based nutritional and hygienic education.  Women in this group will receive additionally:    1. National nutrition and health care including IFA supplementation   2. treatment of any symptomatic health condition and deworming in case of symptomatic complaints during second and third trimesters,   3. Women who experience odor, itching or discharge will be treated for candidiasis and bacterial vaginosis.  Other Name: Video-based counselling |
| Study Arms  ICMJE | - Standard counseling Pregnant women in the control group will receive the standard education package as per the Ethiopian guidelines. In the standard health care, pregnant women receive a minimum of four ante-natal care visits at the health centers during which they also receive iron and folic acid supplementation. They participate in monthly forums facilitated by nurses to answer questions and concerns regarding nutritional care.  Intervention: Behavioral: Standard counselling - Experimental: Health-Video Women in the Health-Video group will receive home visits for delivery of healthy nutrition messages using prepared video-based messages every two weeks. They will also participate in monthly forums facilitated by nurses using also videos for demonstration of nutritional care. During the monthly forums (six in total during the pregnancy and the post-partum periods), the messages will all be given as a video show coordinated by a nurse/ health professional for any questions. During postnatal period, two counseling sessions will be delivered within two weeks of birth, and 12 sessions or twice every month till 6 months.  Intervention: Behavioral: Health-Video |
| Publications \* | - Lassi ZS, Salam RA, Haider BA, Bhutta ZA. Folic acid supplementation during pregnancy for maternal health and pregnancy outcomes. Cochrane Database Syst Rev. 2013 Mar 28;(3):CD006896. doi: 10.1002/14651858.CD006896.pub2. Review. - Rasmussen KM, Stoltzfus RJ. New evidence that iron supplementation during pregnancy improves birth weight: new scientific questions. Am J Clin Nutr. 2003 Oct;78(4):673-4. - Brooker S, Bethony J, Hotez PJ. Human hookworm infection in the 21st century. Adv Parasitol. 2004;58:197-288. Review. - Prociv P, Luke RA. Evidence for larval hypobiosis in Australian strains of Ancylostoma duodenale. Trans R Soc Trop Med Hyg. 1995 Jul-Aug;89(4):379. - Asundep NN, Jolly PE, Carson AP, Turpin CA, Zhang K, Wilson NO, Stiles JK, Tameru B. Effect of Malaria and Geohelminth Infection on Birth Outcomes in Kumasi, Ghana. Int J Trop Dis Health. 2014;4(5):582-594. - Kavle JA, Landry M. Addressing barriers to maternal nutrition in low- and middle-income countries: A review of the evidence and programme implications. Matern Child Nutr. 2018 Jan;14(1). doi: 10.1111/mcn.12508. Epub 2017 Aug 24. Review. - Verstraelen H, Delanghe J, Roelens K, Blot S, Claeys G, Temmerman M. Subclinical iron deficiency is a strong predictor of bacterial vaginosis in early pregnancy. BMC Infect Dis. 2005 Jul 6;5:55. - Tuddenham S, Ghanem KG, Caulfield LE, Rovner AJ, Robinson C, Shivakoti R, Miller R, Burke A, Murphy C, Ravel J, Brotman RM. Associations between dietary micronutrient intake and molecular-Bacterial Vaginosis. Reprod Health. 2019 Oct 22;16(1):151. doi: 10.1186/s12978-019-0814-6. - Ashorn P, Hallamaa L, Allen LH, Ashorn U, Chandrasiri U, Deitchler M, Doyle R, Harjunmaa U, Jorgensen JM, Kamiza S, Klein N, Maleta K, Nkhoma M, Oaks BM, Poelman B, Rogerson SJ, Stewart CP, Zeilani M, Dewey KG. Co-causation of reduced newborn size by maternal undernutrition, infections, and inflammation. Matern Child Nutr. 2018 Jul;14(3):e12585. doi: 10.1111/mcn.12585. Epub 2018 Jan 8. - Bhutta ZA, Das JK, Rizvi A, Gaffey MF, Walker N, Horton S, Webb P, Lartey A, Black RE; Lancet Nutrition Interventions Review Group, the Maternal and Child Nutrition Study Group. Evidence-based interventions for improvement of maternal and child nutrition: what can be done and at what cost? Lancet. 2013 Aug 3;382(9890):452-477. doi: 10.1016/S0140-6736(13)60996-4. Epub 2013 Jun 6. Review. Erratum in: Lancet. 2013 Aug 3;382(9890):396. |
| \*   Includes publications given by the data provider as well as publications identified by ClinicalTrials.gov Identifier (NCT Number) in Medline. | |
|  | |
| Recruitment Information | |
| Recruitment Status  ICMJE | Completed |
| Actual Enrollment  ICMJE    (submitted: October 28, 2020) | 675 |
| Original Estimated Enrollment  ICMJE    (submitted: May 29, 2020) | 580 |
| Actual Study Completion Date  ICMJE | July 31, 2021 |
| Actual Primary Completion Date | July 31, 2021   (Final data collection date for primary outcome measure) |
| Eligibility Criteria  ICMJE | Inclusion Criteria:   - Signed informed consent form - Aged at least 18 years - Permanent resident of the village of the study intervention/control - Planned availability during the whole period of the study (12 months) - Acceptance of the intervention package including home visits for data collection and morbidity follow up.   Exclusion Criteria:   - Severe anemia (hemoglobin <70 g/L), - Under nutrition (defined as body mass index before pregnancy of <18.5 kg/m2), - Chronically ill mothers with tuberculosis or other chronic diseases, - Reported HIV-positive mother. - Individuals with anatomical deformity will be excluded due to the difficulty of measurement of height. |
| Sex/Gender  ICMJE | |  |  | | --- | --- | | Sexes Eligible for Study: | Female | |
| Ages  ICMJE | 18 Years and older   (Adult, Older Adult) |
| Accepts Healthy Volunteers  ICMJE | No |
| Contacts  ICMJE | *Contact information is only displayed when the study is recruiting subjects* |
| Listed Location Countries  ICMJE | Ethiopia |
| Removed Location Countries |  |
|  | |
| Administrative Information | |
| NCT Number  ICMJE | NCT04414527 |
| Other Study ID Numbers  ICMJE | BC-06756 |
| Has Data Monitoring Committee | No |
| U.S. FDA-regulated Product | |  |  | | --- | --- | | Studies a U.S. FDA-regulated Drug Product: | No | | Studies a U.S. FDA-regulated Device Product: | No | |
| IPD Sharing Statement  ICMJE | |  |  | | --- | --- | | Plan to Share IPD: | No | | Plan Description: | All the data that can affect the main or the secondary outcomes will be used in the analyses and shared as necessary.  Data on helminthic infection will use household characteristics and women and infants nutritional status | |
| Current Responsible Party | University Ghent |
| Original Responsible Party | VakgroepVolksgezondheidEnEerstelijnszorg, University Ghent, Professor |
| Current Study Sponsor  ICMJE | University Ghent |
| Original Study Sponsor  ICMJE | *Same as current* |
| Collaborators  ICMJE | - Flemish Interuniversity Council (VLIR) - College of Medicine and Health Sciences, Arba Minch University, Ethiopia |
| Investigators  ICMJE | |  |  |  | | --- | --- | --- | | Principal Investigator: | Stefaan De Henauw, Md. PhD | University of Ghent | | Principal Investigator: | Souheila Abbeddou, MSc. PhD | University of Ghent | | Principal Investigator: | Bruno Levecke, PhD | University of Ghent | |
| PRS Account | University Ghent |
| Verification Date | December 2021 |
| ICMJE     Data element required by the International Committee of Medical Journal Editors and the World Health Organization ICTRP | |

To Top

- For Patients and Families
- For Researchers
- For Study Record Managers

- Home
- RSS Feeds
- Site Map
- Terms and Conditions
- Disclaimer
- Customer Support

- Copyright
- Privacy
- Accessibility
- Viewers and Players
- Freedom of Information Act
- USA.gov
- HHS Vulnerability Disclosure

- U.S. National Library of Medicine
- U.S. National Institutes of Health
- U.S. Department of Health and Human Services
