# Supplementary material for: Acceptability and feasibility of video-based health education for maternal and infant health in Dirashe District, South Ethiopia: A qualitative study
Source: PLOS Glob Public Health. 2023 Jun 29;3(6):e0000821. doi: 10.1371/journal.pgph.0000821 (PMC10309618; doi:10.1371/journal.pgph.0000821)
Supplement: S1 Text — (DOCX) [file pgph.0000821.s003.docx]

**S1 Text: Focus Group Checklist**

**Make arrangements for**

- Private setting for focus group site
- Transportation of staff to focus group site
- Transportation of participants to focus group site
- Refreshments for participants (if applicable)

**What to take to the focus group**

**Equipment**

- 1 tape recorder (plus 1 extra, if available)
- 2 blank 90-minute cassette tapes per focus group
- Spare batteries
- Field notebook and pens
- Name card materials Focus group packet
- 1 large, heavy-duty envelope
- Archival information sheet with archival number
- 2 copies of focus group guide (1 for moderator, 1 for note-taker)
- Informed consent forms (enough for all participants)
- Note-taking form
- Debriefing form
- Participant reimbursement (if applicable)
- Reimbursement form (if applicable)

**What to place in the envelope after the focus group**

- Completed archival information sheet
- Signed inform consent form (signed by moderator and/or note-taker)
- Labeled focus group guide with notes (moderator’s copy)
- Labeled focus group guide (note-taker’s copy)
- Note-taker’s field notes
- Labeled cassette tapes, re-record tabs punched out
- Signed reimbursement form (if applicable)
