# Supplementary material for: Acceptability and feasibility of video-based health education for maternal and infant health in Dirashe District, South Ethiopia: A qualitative study
Source: PLOS Glob Public Health. 2023 Jun 29;3(6):e0000821. doi: 10.1371/journal.pgph.0000821 (PMC10309618; doi:10.1371/journal.pgph.0000821)
Supplement: S2 Text — (DOCX) [file pgph.0000821.s004.docx]

# Data Collection tools

## FGD Guide for mothers in video communities

**Facilitator Name: ______________________**

**Date: _________________________________**

**Woreda: _________________________________**

**Kebele: ______________________________**

**Topic:** Effects of video-based health educations on health and nutritional status

**Overall Objectives:** the aim of this study was to assess the acceptability and feasibility of video-based health education among pregnant and lactating women.

**Background and Introductions (10 MINS)**

**Welcome and Opening Comments**

- Thank participants for agreeing to come.
- Explain what a focus group is.
- Encourage participants to speak freely throughout the discussion.
- Not seeking consensus.
- There are no right or wrong answers.
- Identify topic in broad terms.
- Use the phrase "going to talk about some of the things I mentioned above."
- Tell how long group will last (about 90 minutes).
- Mention that moderator may interrupt to move group along.
- Mention that the session is being audiotaped (and videotaped).
- The information that groups discusses will be analyzed as a whole and will not be analyze individuals independently.
- No participant names will be used in any analysis of the discussion.
- Information given by the group will not be available to the general public.

(NOTE: If a participant asks how the information will be used, the answer *“Effects of video-based health education on health status of pregnant mothers and their infants (from 0 to 6 months) in Dirashe district Southern Ethiopia - A cluster randomized controlled trial.”* should be given).

**Introduction of Participants:**

Could we begin by each of you telling us your first name and a little something about yourself like your responsibility in the community [ask for any employment or involvement in any social duties, Studies achieved]? Let's start with you [address one participant]

1. How do you explain about video-based health project?

**Probe:** access, availability, utilization

1. Would you discuss what a video-based health and nutrition project?

**Probe**: Components

Place where you get the service?

1. What are the videos you seen so far?

**Probe:** the videos projected

1. How do you evaluate the video projections displayed to you in terms of?

**Probe:**

- Clarity
- Understandability
- Waiting time to see the video
- Accessibility
- Affordability
- Respectfulness of cultures, norms, and religion

1. How do you explain the support you have been provided by local research team or video implementers for the projection of the video?

**Probe**:

- Access
- Utilization

1. Can you please discuss what you have learned from the video health project?

**Probe**: New approaches and easy ways you learned

Experiences to be shared to others

1. In your opinion, what were the successes of the video project?

**Probe**: Your expectation

1. What do you suggest on sustainability of the video project?

**Probe**: Integration with the local health care authorities

Scale up

1. How do you explain the limitations/challenges the video-based health education (Health posts and out of health post)?
2. Is there anything else you would like to tell me about regarding the video project?

Thank you for speaking with me today!

## KII tools for mothers with best and/or bad experience

- ***Acceptability:***
  - What was the aspect of the program that you liked the most?
  - What was your favorite session/video?
  - Would you recommend this intervention to someone you care about?
- ***Suggestions for further improvement:***
  - What did you least like about the program?
  - What do you think could be improved about ‘video’?
- ***Barriers/facilitators:***
  - Were there any difficulties to taking part in video?
  - Were there any support or facilitators to taking part in video?
- ***Process of change:***
  - Are there any changes in your perspective of living? If the answer is ‘Yes’, what are they?
  - Have you noticed any differences in your life as a result of taking part in ‘A video-show or display’? If ‘yes’, what are these differences?
  - What did you learn from this program?
- ***Implementing change:***
  - Do you practice lessons learned? How often?
  - Have missed sessions from the display
- ***Feasibility***
  - Any cost incurred to participate in video
  - How much number of people attending video sessions VI

## KII tools for Video implementers

- ***Acceptability:***
  - What was the aspect of the program that you liked the most and also the mothers?
  - What was your favorite session/video your participants liked when you project?
  - How do you judge the clarity and language of the videos displayed in considering the local culture?
  - Would you recommend this intervention to someone you care about?
- ***Suggestions for further improvement:***
  - What did you least like about the program?
  - What do you think could be improved about ‘video’?
  - What aspects you think can be modified for better acceptance and usability?
- ***Barriers/facilitators:***
  - Were there any difficulties for the mothers to taking part in video?
  - Were there any support or facilitators to taking part in video?
  - Are there difficulties from the participants to effective display the video probe: timing, overlapping duties?
- ***Process of change:***
  - Are there any changes in your perspective of living? If the answer is ‘Yes’, what are they?
  - Have you noticed any differences in your life as a result of taking part in ‘A video-show or display’? If ‘yes’, what are these differences?
  - What did you learn from this program?
- ***Implementing change:***
  - Do you think that the messages delivered are easy to practice? How often?
  - Do you faced problems in displaying the videos? Probe: managing the projectors, managing the behavior of mothers, environmental management (selection of appropriate place, avoiding disturbance from other people)
- ***Feasibility***
  - Any cost incurred to participate in video
  - How much number of people attending video sessions VI?
  - How do you the scaling up of the interventions displayed in the videos?

## KII tools for HEWs

- ***Acceptability:***
  - What was the aspect of the program that the mothers liked the most? If you have an experience? Probe: experience sharing during mother’s forum
  - Would you recommend this intervention to someone you care about?
- ***Suggestions for further improvement:***
  - What did you least like about the program?
  - What do you think could be improved about ‘video’?
- ***Barriers/facilitators:***
  - Were there any difficulties to taking part in video?
  - Were there any support or facilitators to taking part in video?
- ***Process of change:***
  - Are there any changes in your perspective of using video? If the answer is ‘Yes’, what are they?
  - Have you noticed any differences in your life as a result of taking part in ‘A video-show or display’? If ‘yes’, what are these differences?
  - What did you learn from this program?
- ***Implementing change:***
  - Do you think that the mothers practice lessons learned? How often?
- ***Feasibility***
  - Any cost incurred to participate in video
  - How much number of people attending video sessions VI
