# Supplementary material for: Acceptability and feasibility of video-based health education for maternal and infant health in Dirashe District, South Ethiopia: A qualitative study
Source: PLOS Glob Public Health. 2023 Jun 29;3(6):e0000821. doi: 10.1371/journal.pgph.0000821 (PMC10309618; doi:10.1371/journal.pgph.0000821)
Supplement: Attachment — Submitted filename: Response to Reviewers.docx [file pgph.0000821.s005.docx]

PGPH-D-22-01021
Acceptability and feasibility of video-based health education for maternal and infant health in Dirashe district, South Ethiopia: A qualitative study
PLOS Global Public Health

Dear Dr. Godana,

Thank you for submitting your manuscript to PLOS Global Public Health. After careful consideration, we feel that it has merit but does not fully meet PLOS Global Public Health’s publication criteria as it currently stands. Therefore, we invite you to submit a revised version of the manuscript that addresses the points raised during the review process.

Please submit your revised manuscript by Oct 23 2022 11:59PM. If you will need more time than this to complete your revisions, please reply to this message or contact the journal office at [globalpubhealth@plos.org](mailto:globalpubhealth@plos.org). When you're ready to submit your revision, log on to <https://www.editorialmanager.com/pgph/> and select the 'Submissions Needing Revision' folder to locate your manuscript file.

Please include the following items when submitting your revised manuscript:

- A rebuttal letter that responds to each point raised by the editor and reviewer(s). You should upload this letter as a separate file labeled 'Response to Reviewers'.
- A marked-up copy of your manuscript that highlights changes made to the original version. You should upload this as a separate file labeled 'Revised Manuscript with Track Changes'.
- An unmarked version of your revised paper without tracked changes. You should upload this as a separate file labeled 'Manuscript'.

Guidelines for resubmitting your figure files are available below the reviewer comments at the end of this letter.

We look forward to receiving your revised manuscript.

Kind regards,

Md Anwarul Azim Majumder, PhD
Academic Editor
PLOS Global Public Health

Dear Dr. Md Anwarul Azim Majumder,

Thank you for your reply sent on September 8, 2022.

Enclosed we are submitting the revised manuscript PGPH-D-22-01021, entitled: “Acceptability and feasibility of video-based health education for maternal and infant health in Dirashe district, South Ethiopia: A qualitative study”

By Wanzahun Godana, Godana Yaya, Kidus Temesgen, Stefaan De Henauw, and Souheila Abbeddou.

We thank the reviewers for their constructive comments, which helped us to improve our paper. Our responses to the reviewers are provided below, and all changes in the manuscript are marked with track changes. We hope that the revisions meet your expectations and that the manuscript will now be acceptable for publication.

Sincerely,

Wanzahun Godana

Journal Requirements:

1. Please amend your detailed Financial Disclosure statement. This is published with the article. It must therefore be completed in full sentences and contain the exact wording you wish to be published.

a. State the initials, alongside each funding source, of each author to receive each grant.
b. State what role the funders took in the study. If the funders had no role in your study, please state: “The funders had no role in study design, data collection and analysis, decision to publish, or preparation of the manuscript.”

If you did not receive any funding for this study, please simply state: “The authors received no specific funding for this work.”

R: The detailed financial disclosure statement has been amended. In the text, now it reads L694-695 “The funders had no role in the study design, data collection and analysis, decision to publish, or the preparation of the manuscript.”

2. Please provide separate figure files in .tif or .eps format only and remove any figures embedded in your manuscript file. Please also ensure that all files are under our size limit of 10MB.

For more information about how to convert your figure files please see our guidelines: <https://journals.plos.org/globalpublichealth/s/figures>

R: Figure 1 is not provided separately and within the regulations of PGPH

3. We noticed that you used "unpublished" in the manuscript. We do not allow these references, as the PLOS data access policy requires that all data be either published with the manuscript or made available in a publicly accessible database. Please amend the supplementary material to include the referenced data or remove the references.

R: Data not shown statement has been dropped in Line 652

4. We have noticed that you have uploaded Supporting Information files, but you have not included a list of legends. Please add a full list of legends for your Supporting Information files after the references list.

R: A list of legends is not added at the end of the references.

5. In the online submission form, you indicated that your data will be submitted to a repository upon acceptance.  We strongly recommend all authors deposit their data before acceptance, as the process can be lengthy and hold up publication timelines. Please note that, though access restrictions are acceptable now, your entire data will need to be made freely accessible if your manuscript is accepted for publication. This policy applies to all data except where public deposition would breach compliance with the protocol approved by your research ethics board. If you are unable to adhere to our open data policy, please kindly revise your statement to explain your reasoning and we will seek the editor's input on an exemption. Please be assured that, once you have provided your new statement, the assessment of your exemption will not hold up the peer review process.

R. the full data used in this qualitative study is accessible at https://data.qdr.syr.edu/dataset.xhtml?persistentId=doi:10.5064/F6MBHFRJ

Reviewers' comments:

Reviewer's Responses to Questions

**Comments to the Author**

1. Does this manuscript meet PLOS Global Public Health’s [publication criteria](https://journals.plos.org/globalpublichealth/s/criteria-for-publication)? Is the manuscript technically sound, and do the data support the conclusions? The manuscript must describe methodologically and ethically rigorous research with conclusions that are appropriately drawn based on the data presented.

Reviewer #1: Yes

Reviewer #2: Yes

2. Has the statistical analysis been performed appropriately and rigorously?

Reviewer #1: Yes

Reviewer #2: N/A

3. Have the authors made all data underlying the findings in their manuscript fully available (please refer to the Data Availability Statement at the start of the manuscript PDF file)?

The [PLOS Data policy](https://journals.plos.org/globalpublichealth/s/data-availability) requires authors to make all data underlying the findings described in their manuscript fully available without restriction, with rare exception. The data should be provided as part of the manuscript or its supporting information, or deposited to a public repository. For example, in addition to summary statistics, the data points behind means, medians and variance measures should be available. If there are restrictions on publicly sharing data—e.g. participant privacy or use of data from a third party—those must be specified.

Reviewer #1: Yes

Reviewer #2: No

4. Is the manuscript presented in an intelligible fashion and written in standard English?

PLOS Global Public Health does not copyedit accepted manuscripts, so the language in submitted articles must be clear, correct, and unambiguous. Any typographical or grammatical errors should be corrected at revision, so please note any specific errors here.

Reviewer #1: Yes

Reviewer #2: Yes

5. Review Comments to the Author

Please use the space provided to explain your answers to the questions above. You may also include additional comments for the author, including concerns about dual publication, research ethics, or publication ethics. (Please upload your review as an attachment if it exceeds 20,000 characters)

Reviewer #1: Congratulations to this very well conducted study. I read the manuscript with interest and can highlight that it is well-structured. The methods have been described in detail and are adequate.
However, I do have one major concern which relates to the results section: This section is far too long, mainly because it consists of very many direct quotes. I suggest to delete some of these quotes and describe the results more in a summary. This is a substantial concern which the authors need to address.

R: We appreciate your kind remarks, and we are pleased that several sections of the manuscript are done adequately. Indeed, we do agree with the reviewer that the result section is long and this is related to the diversity of stakeholders involved (mothers, health development army leaders, Health Extension workers, Nurses, and video Implementers). We have followed the advice of the reviewer and dropped few redundant quotes which have been reflected in other situations. We hope this answers the concern of the reviewer.

Reviewer #2: The article presents acceptability and feasibility of video counseling package for antenatal care, post-pregnancy care for mother and infant in Ethiopia. It appears that this paper presents some part of a trial.
The article needs form refinement and appropriate presentation.
1. Please give some detailed information about the main study/trial and linkage of the qualitative study in the overall study implementation and usage.

R: We thank the reviewer for their comment. The qualitative study was conducted in the context of a parent effectiveness trial. We have addressed this lack of clarity by adding the context of the effectiveness trial in the background (Lines 77-87), and study setting (Lines 106-119).

2. The rationale for using video based counseling in Ethiopian context should be mentioned in the introduction.

R: We have added the rational behind the video-based education in the introduction (Lines 81-87)

3. The specific comments on the manuscript are marked in the document attached.

6. PLOS authors have the option to publish the peer review history of their article ([what does this mean?](https://journals.plos.org/globalpublichealth/s/editorial-and-peer-review-process#loc-peer-review-history)). If published, this will include your full peer review and any attached files.


**Do you want your identity to be public for this peer review?** If you choose “no”, your identity will remain anonymous but your review may still be made public.


For information about this choice, including consent withdrawal, please see our [Privacy Policy](https://www.plos.org/privacy-policy).

Reviewer #1: **Yes:** Florian Fischer

Reviewer #2: **Yes:** Manoja Kumar Das

[NOTE: If reviewer comments were submitted as an attachment file, they will be attached to this email and accessible via the submission site. Please log into your account, locate the manuscript record, and check for the action link "View Attachments". If this link does not appear, there are no attachment files.]

While revising your submission, please upload your figure files to the Preflight Analysis and Conversion Engine (PACE) digital diagnostic tool, <https://pacev2.apexcovantage.com/>. PACE helps ensure that figures meet PLOS requirements. To use PACE, you must first register as a user. Registration is free. Then, login and navigate to the UPLOAD tab, where you will find detailed instructions on how to use the tool. If you encounter any issues or have any questions when using PACE, please email PLOS at [figures@plos.org](mailto:figures@plos.org). Please note that Supporting Information files do not need this step.
