# Supplementary material for: Acceptability and feasibility of video-based health education for maternal and infant health in Dirashe District, South Ethiopia: A qualitative study
Source: PLOS Glob Public Health. 2023 Jun 29;3(6):e0000821. doi: 10.1371/journal.pgph.0000821 (PMC10309618; doi:10.1371/journal.pgph.0000821)
Supplement: Attachment — Submitted filename: Response to reviewers 29032023.docx [file pgph.0000821.s006.docx]

PGPH-D-22-01021R1
Acceptability and feasibility of video-based health education for maternal and infant health in Dirashe district, South Ethiopia: A qualitative study
PLOS Global Public Health

Dear Dr. Godana,

Thank you for submitting your manuscript to PLOS Global Public Health. After careful consideration, we feel that it has merit but does not fully meet PLOS Global Public Health’s publication criteria as it currently stands. Therefore, we invite you to submit a revised version of the manuscript that addresses the points raised during the review process.

The manuscript has been evaluated by ten reviewers, and their comments are available below. Please note that upon resubmission of the revised manuscript, we invited the two previous reviewers but only one of them provided a review. We were therefore required to obtain an additional review from another external reviewer. Unexpectedly, we received eight additional reviews after reaching out to the scientific community.

The reviewers have raised concerns regarding the reporting, methodology and language of this study. Please address all reviewers’ comments where possible. Specifically, we would like to emphasize that several reviewers raised concerns regarding the length of the results section and potentially identifying information about the study participants.

Could you please revise the manuscript to carefully address the concerns raised?

Please submit your revised manuscript by Mar 27 2023 11:59PM. If you will need more time than this to complete your revisions, please reply to this message or contact the journal office at globalpubhealth@plos.org. When you're ready to submit your revision, log on to https://www.editorialmanager.com/pgph/ and select the 'Submissions Needing Revision' folder to locate your manuscript file.
Please include the following items when submitting your revised manuscript:

- A rebuttal letter that responds to each point raised by the editor and reviewer(s). You should upload this letter as a separate file labeled 'Response to Reviewers'.
- A marked-up copy of your manuscript that highlights changes made to the original version. You should upload this as a separate file labeled 'Revised Manuscript with Track Changes'.
- An unmarked version of your revised paper without tracked changes. You should upload this as a separate file labeled 'Manuscript'.

Guidelines for resubmitting your figure files are available below the reviewer comments at the end of this letter.

We look forward to receiving your revised manuscript.

Kind regards,

Johannes Stortz, PhD

Dear Editors

Thank you for the email and feedback regarding our submission of the revised version of our manuscript (PGPH-D-22-01021R1) on February 13^th^, 2023.

We thank the reviewers for their constructive comments, which helped us to improve our paper. Our responses to the reviewers are provided below, and all changes in the manuscript are marked with track changes. We hope that the revisions meet your expectations and that the manuscript will now be acceptable for publication.

Kind regards

Wanzahun Godana, MSc.

Reviewer #1: The manuscript has improved but the major issue which I have pointed to in the last round of reviews still remains: The results section mainly included direct quotations. I am missing a synthesis of results going beyond only putting together the quotes from qualitative interviews. There is more abstraction and interpretation needed.

Response: Dear reviewer, thank you for the very valuable comments. The result section includes a synthesis of the themes and some interpretation. We hope this version meets your expectations.

Reviewer #3: In the sampling, authors talk about mothers with good adherence and poor adherence, how was that defined and based on what information

Response: The definitions are now reported in the main document under operational definitions.

L 196-199: “**Good adherer:** Participating mother who attended at least 75% of the video projections as per their schedule.

**Poor adherer:** Participating mother who attended less than 75% of the video projections as per their schedule.”

In data collection tools...checklists were used....what checklists and for what purpose

Response: checklists were used to correctly collect the data during FGDs and KIIs. We have a separate FGD guide and interview guide for the collection of the data. All were included as a supplementary material.

In result theme 1, the acceptability....were there any issues/challenges on acceptability

Response: Under acceptability, the theme of challenge is not usually included. However, some contents of the video were perceived as a challenge in terms of availability like foods, water. This aspect was discussed under practicality and implementations.

while this was a qualitative study, languages like determinants should be avoided when possible. The objective was to study "assess feasibility and acceptability" but some points discussed are not in line with that

Response: We do agree that the word “determinant” cannot be used in qualitative studies. The change has been made accordingly in the subtitle. Now it reads “Factors affecting acceptability”.

The topic "quantitative measure...." in the discussion is not relevant since the findings are not clearly discussed, more over this is a qualitative study, discussing about quantitative measures is not good scientific practice.

Response: In our main study we defined adherence as quantitative measure that affects the definition of acceptability. However, we agree that this can be misleading and therefore we have re-phrased the term to assessment.

Reviewer #4: Thank you for the opportunity to review this qualitative study on the acceptability and feasibility of video-based health education for maternal and infant health in South Ethiopia. It was an interesting topic to read about and was well structured.

We thank the reviewer for your positive feedback.

1. In as much as a summary of the main study implementation has been added to the manuscript, it will benefit from a brief summary of how the videos were shown during the study. From the results section, several scenarios are alluded to: videos shown individually and in groups, at home (or other venues in the community) and in the clinic. It will therefore be easier to contextualize the quotes if there is a summary of how the videos were shown during the course of the study or at the very least, how they were intended to have been shown in the Study setting sub-section.

Response: The implementation is now detailed in the text (L123-124), which reads as “. The videos were projected every two weeks at the home of the participating women, and monthly in forum or in group, at nearby health facility, church or at the school.”

2. It may be beneficial to briefly explain how ‘adherence’ was defined to determine who qualified as having good or low adherence in the operational definitions section.

Response: the definition is now included

3. Line 276 “Mothers unanimously liked the videos, as they are linked to their health.” However, the quote given was about what the women’s least favourite video was. You may want to consider using a quote that will better reflect the sub-theme of what was unanimously liked (or change the sub-theme to what the mothers did not like about the videos and present the quote(s) to buttress this sub-them).

Response: Thank you for this pertinent comment. Now corrected and the quote given has been replaced to “The women said they liked most of the videos but loved the video of giving birth in a health facility. Because, they say that they can be taken care of in an ambulance immediately in case of emergency and this is a lifesaving.”

4. In the discussion section, you gave a hypothesis which is the foundation of your discussion (lines 528-585). Please add the citation(s) for this hypothesis.

Response: we included a text in the introduction section of the article regarding the hypothesis.

5. A couple of the themes in the Figure 1 were not really explored in the results or the discussion: political environment and empathy of implementers. It will be beneficial to explore these themes further in the manuscript if they were indeed themes that emerged or justify their presence in the figure.

Response: Thank you for raising very important point. For political environment: it is to show the shortage of budgets to continue and to show the videos for many of the mothers. Maybe it would be better to change the word to government support.

6. A minor point: A few quotes had full participant names attached to them, for example, on line 381. Better to give only the 1st name as is best practice and has been done throughout the paper. On the same line, participant was also identified as “FGD” without a description of what their role was (FGD discussant?).

Response: All the identifiers have been dropped and the quotes are anonymized.

Reviewer #5: This qualitative study demonstrated acceptability and feasibility of video-based health education intervention for maternal and infant health through FGDs and KIIs among different stakeholders in a district in South Ethiopia.

1) Although the authors tried to address the issues highlighted in the first review, the major concerns related to length of result section remains yet. I would like to suggest author to interpret the results more precisely, particularly for the long quotes.

Response: The balance between providing the details and the length of the text is indeed challenging. In this version, we added more description of the results that can guide the logic behind the quotes. We hope that this answers your concern.

2) Author should be specific about their intervention. They mentioned intervention in different terms like “community-based video intervention”, “video-based intervention”, or “video-based health education”. It should be consistent in whole manuscript. It seems to be a video-based health education intervention, suggested to replace by it.

Response: Despite the inconsistency in the terms used, we did mean the same thing. All the terms were harmonized now all along the text.

3) Please use full-form when you mentioned time (e.g., 1 hour 25 minutes to 1 hour 45 minutes in line 167, 35 minutes to 55 minutes in line 168).

Response: corrected as suggested.

4) There are several typo-errors (e.g., 37 KIIs in line 23, 10% in line 175, and so on), please check the whole manuscript.

Response: we checked for the typo-errors and corrected it. For the 10% in line 175, is for quality control only. Randomly checked 10% was 5 transcripts from 46 transcripts to check whether the ascriptions and translation was correct.

5) Please check reference styles, few references seem to be inconsistent. Author used both full-form and abbreviation of journal name.

Response: corrected as per the comment and references are now harmonized

Reviewer #6: 1. On L28 and 32the word “good” needs to be clarified.

Response: Both sentences have been re-phrased to increase clarity to “the video-based health education intervention was acceptable and feasible.”

2. Please remove L151-153- you don’t need to quantify qualitative research.

Response: We are not sure what the reviewer means here. This section was not provided with the aim to quantify the research. However, it is to define the concepts so that the readers can easily understand it.

3. The result section still needs trimming and ramifications.

Response: We have modified this section to minimize the lengthy quotes, and we included also description of the different themes/quotes.

4. Please interpret the data and put a quote that supports the point you wanted to make. Please select a single quote from one study group that can clearly elaborate on the idea you want to raise.

Response: We agree with the reviewer in this point in using a single quote to elaborate our results as well as enriching more the description of the results. However, in some situations, we have opted to add multiple quotes as recommended by Heery et al. (2019) who indicate that verbatim quotes enhance the confirmability of the findings.

5. Walayte (FGD discussant)- Please correct the name of the place.

Response: corrected as per the comment

6. Please use a similar labelling format for the quotes. E. g (HDA), (FGD discussant), (FGD) Mention either the study type, the respondent type or both uniformly.

Response: Thank you for raising this issue of identifier. We were asked in similar way when publishing our data in data repository and we corrected accordingly.

7. Please remove personal identifiers – e.g “Kolla Mashile” if these are names of a district, please mention the district names in the methods section.

Response: corrected as per the comment

8. Please reduce the length of some lengthy quotes. Go for a maximum of 6 lines.

Response: We appreciate that some quotes were long. However, some times it maybe affect the readability and better to keep it . For example, Corden and Sainsbury (2006) suggest that researchers quote for providing explanations, and to elucidate processes and informants’ perspectives. Further, quotations are also sometimes used as illustration; for example, a need to describe how something has affected an informant is illustrated by means of presenting his or her own words. Corden and Sainsbury (2006) even suggest that quoting deepens the understanding by means of enrolling the informant’s voice. All in all, quotations are supposed to enhance readability, providing vividness to the text. Thus, quotations aid communication. Accordingly, quotations also serve as evidence: original data help to serve the reader’s assessment of the accuracy of the analysis, thereby strengthening the findings.

However, we tried to minimize based on the comments.

Reviewer #7: The manuscript presents a qualitative study conducted at the end of a Cluster Randomized Community Trial. It is an appropriate complement to the main study as it endeavors to evaluate the intervention implemented.

We thank the reviewer for rightfully putting this study in the context of the parent study. We will address the comments in the next sections.

On the whole, study focus, logical flow and cohesion of the paper needs improvement. General areas of improvement are as follows:

1. Study focus

To enhance understanding and appreciation of the manuscript, the focus/objectives of this qualitative study need to be made very clear in the introduction and run through the other sections of the write up (methodology, results, discussion and conclusion) to ensure logical flow of the manuscript. The variables of acceptability and feasibility as well as the methods and tools of their assessment also need to be explicitly stated. Differing objectives and variables are alluded to in different sections of the manuscript making it challenging to discern the core study focus.

Response: the context of the study might have been missing indeed. We have provided the focus of the study now in the introduction and the discussion.

2. Introduction
This should be shortened to succinctly make a case for the qualitative study in relation to why digital innovative methods to facilitate nutrition education and counselling need to be in place
• Importance of looking at the feasibility and acceptability of the video-based health education methods and their linkage to promoting behavior change at scale

• The limitation of information provided in ANC.

Response: We understand that the introduction was quite lengthy. However, the context is specific to the Ethiopia health system, and we felt that there is a need to explain how the system functions. Otherwise, it will be hard to understand why alternative methods for communication in behavior change are needed. However, we have dropped the part of nutritional education and its effects.

Furthermore, the last paragraph of the introduction section describes the importance of acceptability. Lines 81-82 “….the importance given by the community and the health workers(18).”

Regarding the limitations, lines 87-89 “Traditional methods of dissemination such as pamphlets and flipcharts exclude people with low literacy and are not environmentally conscious. Fatigue over repeated messages is also a concern in behavior change interventions” provide this information

It is unclear what beliefs, attitudes, needs and situations were being investigated. Furthermore, aspects of identifying opportunities and challenges are also referred to but results related to this are not reflected in the abstract nor results section of the paper. Study focus needs to be appropriately synchronized in the title and main body of the paper.

Response: Thank you for the comment. However, the challenges of the program were clearly stated under results section. The sub section was barriers and challenges. L362-414. The opportunities were ease of application, benefits and acceptance which reflects the opportunities and where it can be used as alternative health education method.

3. Materials and methods

Expound on specific procedures, tools and variables of interest for the qualitative study.

Response: The study procedures were detailed including those related to the parent study. Therefore, we are not clear where more details are needed

4. Results
   • Provide evidence-based results from the FGDs and KIs that are well linked to the objectives and variables of interest
   • Need to standardize labelling of quotes by location, data collection method and respondent type.
   • Ensure privacy and confidentiality of respondent is safeguarded

Response: thorough revision has been made, which we hope that it meets the expectations of the reviewer. Privacy of the participants was now assured by anonymizing the identifies

5. Discussion should
• be closely linked to the result
• logically flow as per the objectives/results presented without intermingling different issues
• show linkage or difference with similar studies done in the same or different context.

Response: the results section was modified and linked with the discussion

6. Conclusion and implications
• Conclusions should be drawn from the study results
• Implication needs to allude to if acceptability and feasibility was good, what does that mean for maternal and infant health
Response: The implication of the study was included as it can be seen as alternative health information dissemination system that is acceptable and whose implementation is feasible. This was the aim of the qualitative study. We cannot at this stage use further the effectiveness results as these are still under analysis and full conclusions cannot be drawn before the main outcomes are analyzed

7. Check grammar and sentence construction ensuring logical connection between the sentences and paragraphs.

Response: Thank you. The English was checked additionally using <https://www.scribendi.com/>

Reviewer #8: Based on the findings in the research, I agree with the authors claim that the acceptability and feasibility of video-based health education for maternal and infants in Dirashe district is good. I think that the results are strongly supported with the data provided.
I only have minor edits (in tract changes) and comments. I would prefer to reduce the quotations in each theme. About two or maximum of three should be enough to support a theme. For example, in line 288- 'Feasibility of video-based health education among pregnant and lactating women...': there are five quotations which could be reduced to two or a maximum of three of different participants.
My overall impression is that the paper is strong and i recommend for it to be published with these minor edits/comments.

Response: We thank the reviewer for the very positive feedback. We have considered the shortening of the quotations as advised.

Reviewer #9: This significant research project uses a phenomenological qualitative study design to examine the feasibility and acceptability of video-based behavioral change communication in the local setting with the goal of enhancing both the health of the mother and the newborn Overall, the manuscript is highly fascinating, straightforward, and condensed. Address the aforementioned suggestions to make the manuscript more valuable.

Response: Thank you for your very positive evaluation. We have addressed all the comments to improve the manuscript.
